# Malaria impact on cognitive function of children in a peri-urban community in the Brazilian Amazon

**DOI:** 10.1186/s12936-019-2802-2

**Published:** 2019-05-16

**Authors:** Raquel Tapajós, Daniel Castro, Gisely Melo, Seyi Balogun, Mark James, Rockson Pessoa, Anne Almeida, Mônica Costa, Rosemary Pinto, Bernardino Albuquerque, Wuelton Monteiro, José Braga, Marcus Lacerda, Maria Paula Mourão

**Affiliations:** 1 Fundação de Vigilância em Saúde do Amazonas, Manaus, Brazil; 2 Fundação de Medicina Tropical Dr. Heitor Vieira Dourado, Manaus, Brazil; 3 Escola Nacional de Saúde Pública Sérgio Arouca, FIOCRUZ, Rio de Janeiro, Brazil; 4 Escola Superior de Ciências da Saúde, Universidade do Estado do Amazonas, Manaus, Brazil; 5 Instituto de Medicina Social, Universidade do Estado do Rio de Janeiro, Rio de Janeiro, Brazil; 6 Instituto Leônidas e Maria Deane, FIOCRUZ, Manaus, Brazil; 7 Kent University, Kent, OH USA

**Keywords:** Malaria, *Plasmodium vivax*, Cognition, Children

## Abstract

**Background:**

In Latin America, where *Plasmodium vivax* malaria is more prevalent, it is known that this species plays an important role in the sustainability of transmission, and can have an impact on morbidity in terms of anaemia, nutritional status, and cognitive development in children.

**Methods:**

The present study aimed to assess the impact of malaria infection on cognition of children in a peri-urban community in the Brazilian Amazon with moderate endemicity by applying Home Inventory and WPPSI-IV. A non-concurrent cohort study was designed and the cognitive, haematological, and nutritional profiles of the children were assessed. Children with documented malaria history were identified from official reported data.

**Results:**

A total of 219 children aged between 2 and 7 years were enrolled. Although 205 (95%) children had normal birth weight, 177 (81%) were malnourished, and 35 (16%) had anaemia. Among the 100 (46%) children who experienced at least one episode of malaria, 89 (89%) children demonstrated low level of cognitive development. The findings showed that *Plasmodium vivax* malaria was an independent risk factor for low cognitive development.

**Conclusions:**

In addition to the known economic impact of malaria in the Amazon region, the study highlights the deleterious effects *P. vivax* malaria has on the socio-cultural development of the population.

## Background

Malaria is the second most common cause of infectious disease-related death in the world. It is estimated to affect 210 million people annually and accounts for approximately 450,000 deaths per year [1].

In the Americas, Brazil reports most of the morbidity attributed to malaria, with the disease being responsible for a substantial decrease in the quality of life [2, 3]. There have been several malaria control efforts in Brazil; nonetheless, children are still greatly affected [4]. Most children living in endemic areas suffer multiple episodes of malaria prior to adolescence. In general, these episodes are acute, not complicated, and children recover after treatment, especially in areas where *Plasmodium vivax* is frequent [5, 6]. Studies examining neurological sequelae in children with severe falciparum malaria (mostly cerebral malaria) have shown that there is some impairment in developing cognitive abilities after an acute episode, both in the short-term [7–9] and long-term [10, 11]. Greater clarification on the effects of cumulative or repetitive malaria episodes on cognitive development of children is needed, in particular that caused by *P. vivax*.

Considering that *P. vivax* is more prevalent than *Plasmodium falciparum* in Brazil [2] and that previous study, also in the Brazilian Amazon, showing that malaria compromises the school performance of children [12], the principal aim the present study was to assess the impact of malaria on cognitive function in children who live in the *Brasileirinho*, a small peri-urban community on the eastern outskirts of Manaus, Brazil.

## Methods

### Ethics statement

The study was approved by the Ethics Review Board of *Fundação de Medicina Tropical Dr Heitor Vieira Dourado* (FMT-HVD) (approval number 1.066.733/2015). Both the Municipal Secretaria of Education and school directors also approved the study. Parents/guardians signed an informed consent.

### Study area and population

The city of Manaus has a population of 2,020,301 inhabitants, mostly living in urban or suburban areas of the city [13]. In this municipality, intense migratory processes, combined with poor surveillance and entomological detection, results in active malaria transmission in rural and peri-urban areas.

**Fig. 1:**
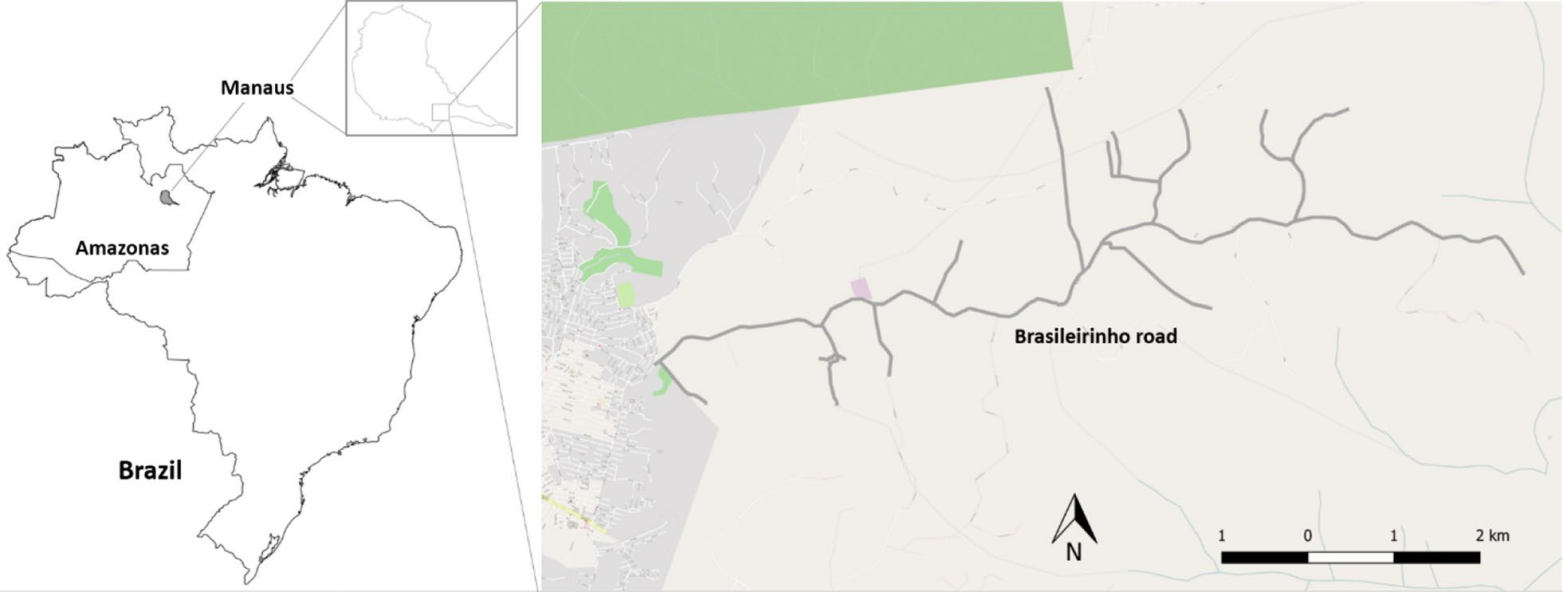
Map of *Brasileirinho*, Manaus, Amazonas

The study site, *Brasileirinho*, is a community with a reported population of 1574 inhabitants, located in the peri-urban region of eastern Manaus (Fig. 1). At this site, residents are vulnerable to malaria transmission due to standing water and ponds used as community bathing pools. The occurrence of malaria is common in this population since water for sanitation and consumption comes from wells or streams that serve as mosquito breeding sites. This community has access to one malaria clinic specifically conducting malaria diagnosis by microscopy and treatment of cases. Health agents based in this clinic regularly visit homes in order to ensure early detection and treatment of cases.

### Study design

Cross-sectional surveys were conducted in the community, including all inhabitants that were willing to participate in the study. In case of absence of any family members, the team returned to the respective house for a second time. All children living in the area were identified. The community was sensitized and informed about the study and participants were invited to participate.

Inclusion criteria consisted of: age between 2 years and 6 months old (30 months) and 7 years and 7 months old (91 months), and residence in the study area. Exclusion criteria consisted of: presence of reported co-morbidities that are known to affect cognitive development (Autism Spectrum Disorder [ASD], Attention Deficit Disorder Association [ADHD], and other neurological diseases), severe episodes of acute infectious diseases in the last 30 days and/or presence of malaria parasites in peripheral blood during screening. After obtaining consent, a questionnaire was administered to survey participant information about the history of recent illness, socio-demographic data were collected. The history of malaria was confirmed and obtained through of the Information System for Epidemiological Surveillance and Notification of Malaria Cases (SIVEP-Malaria/SVS/MS). This system, the results of the microscopy are typed and stored. All children were positive for *P. vivax.*

A volume of 300 µL of blood was collected by finger puncture using Microtainer^®^ tubes containing EDTA and sodium fluoride (Becton–Dickinson, NJ, USA).

### Diagnosis of malaria

Thick blood smears and PCR was performed to confirm negative diagnosis at the time of cognitive evaluation. Thick blood smears were Giemsa-stained according to World Health Organization guidelines [14]. Numbers of asexual blood stage parasites and gametocytes were determined per 200 leukocytes and parasites per μL were calculated using an assumed density of 6000 leukocytes per μL [15]. The reading of the thick blood smears were performed by two trained microscopists (double blind reading). For genomic DNA extraction, a FavorPrep™ 96-well Genomic DNA Kit (Favorgen, Ping-Tung, Taiwan) was used, according to the manufacturer’s instructions. Qmal Taqman PCR was used to detect *Plasmodium* spp. in DNA samples, as described by Almeida et al. [16].

### Cognitive assessment

An evaluation of cognitive function was conducted to investigate the effect of malaria episodes on cognitive development, taking into account malarial history. Cognitive assessment data were collected at the children’s residence or school. Space was provided for the child to carry out the activities proposed by each evaluation instrument. Data collection was carried out by psychologists, trained in the use of cognitive assessment tools. This assessment allowed the development of more complex and conclusive causal models in the role of infection on the child’s cognitive functions.

Proper guidance by the research team on how to seek specialized care was given to caregivers of children who were diagnosed with cognitive impairment or severe malnutrition.

### Instruments used

#### WPPSI-IV

Wechsler Preschool and Primary Scale of Intelligence–IV (WPPSI-IV) [17] allows evaluation of children beginning at age 2 years and 6 months until the limit of 7 years and 7 months of age. The test is known to have good correlation with other tests that evaluate cognitive development, such as Wechsler Intelligence Scale for Children (WISC). It consists of a battery of tasks to be performed by the child, grouped into two large blocks, executive and verbal, that enable the evaluation of different areas of cognition, summarized in the end by an overall score. A total of four cognitive sub-tests were completed with children ages 2 years, 6 months to 3 years, 11 months (verbal comprehension, working memory, visual spatial, and full scale); and six tests on children ages 4 years, to 7 years, 7 months (fluid reasoning and processing speed, in addition to the previously stated tests). The full-scale composite score is derived from the four or six subtests, and summarizes the ability across a diverse set of cognitive functions, such as verbal comprehension, working memory and processing speed. This score is considered the most representative indicator of global intellectual functioning. The full-scale scores for the analysis of cognition was used.

Although the WPPSI-IV scale has not yet been validated for the Brazilian children, previous research [18] of this, as well as the WISC scale (Weschler Scale for Children) were used in the Brazilian population and showed good reliability and resulting similar to that found in this study.

#### Home inventory

The Resource Inventory Home Environment (RAF) evaluates the quality of the home environment in the first years of life [19]. HOME Inventory assesses resources of the family environment that could contribute to learning in three domains: resources that promote proximal processes; activities that signal stability in family life; and parenting practices that promote family-school connection. This version consists of ten questions that correspond to resource areas previously identified.

The resource area that promotes proximal processes, stimulates participation in developmental experiences such as tours and travel; opportunities to interact with parents; availability of toys and materials that present challenge to thinking; availability of books, newspapers and magazines; proper use of free time; and access to scheduled learning activities. Routines and regular family meetings, and child cooperation housekeeping chores, are included in the activities domain and signal stability in family life. Finally, parental practices that promote bonding domain, e.g., family-school involvement including direct indicators of parents in school life, and participation in teacher-parent meetings for follow-up of academic performance (grades earned). The HOME Inventory was carried out to see if other factors within the home may have a confounding effect on cognition.

### Nutritional assessment

Weight and height were measured by internationally accepted methods [20]. Weight was measured by use of a digital scale, and height was assessed by a single observer with the use of a tape measure. Body Mass Index (BMI) was calculated using the program EPI-INFO version 7.0. BMI Z-score < -2 was defined as malnutrition; scores between − 2 and Z < -1 as underweight (risk zone); Z score between − 1 and 2, normal weight; and Z score > 2 as obesity.

### Haemoglobin

Haemoglobin concentration was measured in venous blood obtained from digital puncture, using a portable HemoCue^®^ photometer (Anglholm, Sweden).

### Stool examination

Stool samples from children included in the study were collected. Analysis of the faecal material was performed using Kato-Katz method, which allows the identification and quantification (number/g of faeces) of a certain helminth infection (*Ascaris lumbricoides*, hookworm, *Schistosoma mansoni*, *Trichuris trichiura*, *Taenia* sp, *Enterobius vermicularis* and *Strongyloides stercoralis*).

### Analytical procedures

A model of hierarchical analysis was developed to determine the influence of the home environment, socio-economic characteristics of caregivers, child nutritional status, and the effect of intestinal helminthiasis on cognitive development measures. The variables were arranged in hierarchical blocks according to their nature and impact on the outcome studied.

Socio-economic status was measured, based on the following variables: (1) household income (families with income equal to or less than 1 minimum wage); (2) running water (families that have water piped into their homes); (3) sewer (families that have network sewage disposal); and (4) home density (number of residents in the house).

The child’s health status was assessed, based on variables relating to: (1) malnutrition (measured by height for age and weight by age, according to the standard adopted by the World Health Organization); (2) anemia (in which children with a haemoglobin concentration < 11.0 g/dL are considered anaemic); (3) intestinal parasites (occurrence of intestinal parasitosis and species found in faecal specimens); (4) low birth weight (based on health registry, considering neonates weighing less than 2500 g at birth); (5) premature birth (measured by records of gestational age at birth, considering those born before the 37th week of gestation); (6) breastfeeding practices (exclusive breastfeeding for at least the first 6 months of life); (7) vaccination (measured by the child’s health records); (8) infectious diseases (occurrence of infectious diseases in the last 30 days prior to the cognitive assessment); and (9) chronic diseases (presence of chronic disease in the child’s medical history).

Organization of the family unit was evaluated by the following variables: (1) adolescent mother (mothers under 18 years of age); (2) mother’s educational level (at least a year of study as reported by mother); (3) family stimulus (measured by the HOME Inventory information, considering four categories according to the final score inventory: more stimulated, regularly stimulated, little stimulated, almost no stimulus); (4) disorders during pregnancy (measured by information relating to the diagnosis of infectious diseases during pregnancy).

Information related to the presence of malaria was evaluated using the following variables: (1) malaria history (at least one prior malaria episode in the child); (2) number of episodes (number of malaria episodes); (3) age/malaria (age at the last episode of malaria); (4) exposure to malaria (measured according to four categories: no exposure—never had malaria; mild—had one episode at 5 years or older; moderate—had one episode prior to 2 years of age, or more than one episode after 2 years of age; severe—had at least two episodes prior to 2 years of age); (5) severe malaria and hospitalization (if there was hospitalization during the malaria episode); (6) malaria and breast feeding (occurrence of malaria episode during the 6-month breast feeding period); and (7) malaria and pregnancy (occurrence of malaria attacks during pregnancy that led to the child being studied).

### Data analysis

Descriptive statistical analyses were performed to characterize the socio-demographic characteristics of children in relation to the level of cognitive development. Different scores of cognitive impairment were used in children to improve understanding of the effect of this disease state on cognitive development.

Associations were tested between cognitive performance and each of the risk factors by logistic regression analysis, assuming a 5% significance level. To better understand the multiplicity of cognitive development, the variables were organized by blocks, according to its nature and impact on the outcome.

Associations with statistical significance in univariate analysis were tested, using a model of hierarchical logistic regression. A step-wise procedure for selecting variables in the multivariate analysis was used. The final model was developed using only the variables that presented an association with the outcome at a 5% significance level. All statistical tests were performed with Stata (Stata Corp, College Station, Texas, USA).

## Results

Initially, 263 children were available in the area study and were screened for the presence of malaria parasites. Ten children who were PCR-positive for *Plasmodium vivax* and 34 children failed to perform the cognition test within the time provided, in accordance with WPPSI-IV recommendations were excluded. A total of 219 children, ages 2–7 years old, were evaluated in a non-concurrent cohort study allowing for the observation of malaria exposure status and its effect on cognitive function.

A total of 219 students aged between 2 and 7 years ($\overline{x}$ = 4 years of age) were enrolled; 114 (52%) male, and 105 (48%) female. Of this total, 97 (44%) were students. Regarding socioeconomic status, 20 (9%) families received less than 1 minimum wage; most residences did not have piped water (60%) or sewage system (98%), and the houses presented with high household crowding (26%). In the group of maternal and family conditions, 119 (50%) children had little or almost no family stimulus, and 198 (91%) of the mothers lacked education. Although 205 (95%) children had normal birth weight, 177 (81%) were malnourished, and 35 (16%) had anaemia. Among the 100 (46%) children who experienced at least one episode of malaria, 89 (89%) children demonstrated low level of cognitive development (Table 1).

**Table 1:** Characteristics of children studied according to the level of cognitive development

Characteristic	No low cognitive development		Low cognitive development		Total	
	N	%	N	%	N	%
Demographic						
Sex						
Male	16	14.0	98	86.0	114	100.0
Female	19	18.1	86	81.9	105	100.0
Total	35		184		219	
Age						
2.6–3.11	15	21.1	56	78.9	71	100.0
4–7.7	20	13.5	128	86.5	148	100.0
Total	35		184		219	
Education						
Not study	24	19.7	98	80.3	122	100.0
Study	11	11.3	86	88.7	97	100.0
Total	35		184		219	
Socio-economic						
Brick-built house						
No	23	16.4	117	83.6	140	100.0
Yes	12	15.2	67	84.8	79	100.0
Total	35		184		219	
Family income						
≤ 1 min. wage	3	15.0	17	85.0	20	100.0
> 1 min. wage	32	16.1	167	83.9	199	100.0
Total	35		184		219	
Piped water						
No	28	21.2	104	78.8	132	100.0
Yes	7	8.0	80	92.0	87	100.0
Total	35		184		219	
Sewage system						
No	34	15.8	181	84.2	215	100.0
Yes	1	25.0	3	75.0	4	100.0
Total	35		184		219	
Housing conditions						
Precarious	7	15.2	39	84.8	46	100.0
Reasonable	21	22.1	74	77.9	95	100.0
Better conditions	7	9.0	71	91.0	78	
Total	35		184		219	
> 2 residents/room						
No	27	16.7	135	83.3	162	100.0
Yes	8	14.0	49	86.0	57	100.0
Total	35		184		219	
Maternal and family conditions						
Adolescent mother						
No	34	15.7	183	84.3	217	100.0
Yes	1	50.0	1	50.0	2	100.0
Total	35		184		219	
Mother’s education						
Not study	31	15.7	167	84.3	198	100.0
Study	4	19.0	17	81.0	21	100.0
Total	35		184		219	
Disorders during pregnancy						
No	33	16.9	162	83.1	195	100.0
Yes	2	8.3	22	91.7	24	100.0
Total	35		184		219	
Level of family stimulus						
More stimulated	5	9.1	50	90.9	55	100.0
Regularly stimulated	9	16.4	46	83.6	55	100.0
Little stimulated	12	21.1	45	78.9	57	100.0
Almost no stimulus	9	17.3	43	82.7	52	100.0
Total	35		184		219	
Child health						
Malnutrition						
No	8	19.0	34	81.0	42	100.0
Yes	27	15.3	150	84.7	177	100.0
Total	35		184		219	
Anemia						
No	32	17.4	152	82.6	184	100.0
Yes	3	8.6	32	91.4	35	100.0
Total	35		184		219	
Intestinal parasites						
No	6	16.7	30	83.3	36	100.0
Yes	29	15.8	154	84.2	183	100.0
Total	35		184		219	
Low birth weight						
No	33	16.1	172	83.9	205	100.0
Yes	2	16.7	10	83.3	12	100.0
Total	35		182		217	
Premature birth						
No	32	15.5	174	84.5	206	100.0
Yes	3	23.1	10	76.9	13	100.0
Total	35		184		219	
Breast feeding until the sixth month						
No	12	14.0	74	86.0	86	100.0
Yes	12	16.4	61	83.6	73	100.0
Total	24		135		159	
Vaccinated						
No	1	4.5	21	95.5	22	100.0
Yes	34	17.4	161	82.6	195	100.0
Total	35		182		217	
Infectious disease in the last 30 days						
No	34	15.8	181	84.2	215	100.0
Yes	1	25.0	3	75.0	4	100.0
Total	35		184		219	
Chronic diseases						
No	34	15.8	181	84.2	215	100.0
Yes	1	25.0	3	75.0	4	100.0
Total	35		184		219	
Exposure to malaria						
Occurrence of malaria						
No malaria history	24	20.2	95	79.8	119	100.0
Had an episode/more	11	11.0	89	89.0	100	100.0
Total	35		184		219	
Number of malaria episodes						
1	7	10.8	58	89.2	65	100.0
2	1	8.3	11	91.7	12	100.0
3	2	14.3	12	85.7	14	100.0
4	1	11.1	8	88.9	9	100.0
Total	11		89		100	
Exposure to malaria						
No exposure	24	20.2	95	79.8	119	100.0
Mild	1	9.1	10	90.9	11	100.0
Moderate	8	10.8	66	89.2	74	100.0
Severe	2	13.3	13	86.7	15	
Total	35		184		219	
Malaria-related hospitalization						
No	9	9.8	83	90.2	92	100.0
Yes	1	25.0	3	75.0	4	100.0
Total	10		86		96	
Malaria during pregnancy						
No	30	15.5	164	84.5	194	100.0
Yes	5	20.0	20	80.0	25	100.0
Total	35		184		219	
Malaria attacks during pregnancy						
No	9	9.8	83	90.2	92	100.0
Yes	1	25.0	3	75.0	4	100.0
Total	10		86		96	

Children who had had at least one episode of malaria had lower mean scores for each of the dimensions assessed in WPPSI-IV compared to those who had never had malaria (Table 2). It is noteworthy that the scores of the dimensions of the WPPSI scale obtained in this study showed an asymmetric distribution with a high proportion of children with low scores. For the purposes of analysis, it was determined that children with a final score of 59.65 and lower (25% percentile) were considered with low cognitive development.

**Table 2:** Cognitive assessment dimensions of exposure to malaria

Variable	Groups of children (n)	Average score	SD of score	Minimum score	Maximum score	*P* value
ICV—Verbal comprehension index	Without malaria (n = 119)	65.3	17.7	45	138	0.004
	With malaria (n = 100)	59.5	13.5	45	111	
IOP—Perceptive organization index	Without malaria (n = 119)	69.0	18.0	45	129	0.0005
	With malaria (n = 100)	61.6	14.4	42	109	
IMT—Working memory índex	Without malaria (n = 119)	69.8	17.7	45	113	0.0055
	With malaria (n = 100)	63.7	17.4	45	126	
CIT—Total intelligence quotient	Without malaria (n = 119)	66.4	21.4	40	155	0.0087
	With malaria (n = 100)	60.3	14.9	40	117	
IVP—Processing speed index	Without malaria (n = 119)	69.7	17.2	45	115	0.0544
	With malaria (n = 100)	65.4	15.7	43	109	
IRA—Abstract reasoning index	Without malaria (n = 119)	67.4	15.1	43	100	0.0135
	With malaria	62.0	14.5	43	114	

Bivariate analysis using logistic regression models showed that children with anemia (OR = 2.24; IC 95% 0.64–7.78), and who had had one episode or more of malaria (OR = 2; IC 95% 0.95–4.41) were more likely to have low cognitive development. None of the variables related to maternal and family conditions were associated with the outcome at the 5% significance level in the bivariate logistic regression model. With multiple regression analysis, associations between low cognitive development and children with anemia (OR = 2.9; IC 95% 0.81–10.22), and occurrence of an episode or more of malaria (OR = 2.2; IC 95% 1–4.85) remained statistically significant (Table 3). These factors showed a greater magnitude in the measure of association.

**Table 3:** Factors associated with low level of cognitive development using univariate and multivariate hierarchical level analysis

Factors	%	OR	CI 95%	OR adjusted	CI 95%
Socio-economic					
Brick-built house					
No	63.9	1			
Yes	36.1	1097	0.513–2.346		
Family income					
≤ 1 min. wage	9.1	1			
> 1 min. wage	90.9	0.92	0.254–3.326		
Sewage system					
No	98.2	1			
Yes	1.8	0.563	0.569–5.579		
Housing conditions					
Precarious	21	1			
Reasonable	43.4	0.632	0.247–1.617		
Better conditions	35.6	1820	0.595–5.569		
> 2 residents/room					
No	73.9	1			
Yes	26.1	1.225	0.521–2.877		
Maternal and family conditions					
Adolescent mother					
No	99.1	1			
Yes	0.1	0	0.011–3.042		
Mother’s education					
Not study	90.4	1			
Study	9.6	1	0.248–2.503		
Disorders during pregnancy					
No	89.1	1			
Yes	10.9	2240	0.502–9.993		
Level of family stimulus					
More stimulated	25.1	1			
Regularly stimulated	25.1	0.511	0.159–1.637		
Little stimulated	26.1	0.375	0.122–1.147		
Almost no stimulus	23.7	0.477	0.148–1.534		
Child health					
Malnutrition					
No	19.2	1			
Yes	80.8	1307	0.543–3.127		
Anemia					
No	84.1	1		1	
Yes	15.9	2245	0.647–7.785	2.877	0.810–10.221
Intestinal parasites					
No	16.4	1			
Yes	83.6	1.062	0.405–2.779		
Premature birth					
No	94.1	1			
Yes	5.9	0.613	0.159–2.350		
Low birth weight					
No	94.5	1			
Yes	5.5	0.959	0.200–4.579		
Breastfeeding until the sixth month					
No	54.1	1			
Yes	45.9	0.824	0.345–1.965		
Vaccinated					
No	10.1	1			
Yes	89.9	0.225	0.029–1.734		
Infectious disease in the last 30 days					
No	98.2	1			
Yes	1.8	0.563	0.569–5.579		
Chronic diseases					
No	98.2	1			
Yes	1.8	0.563	0.569–5.579		
Exposure to malaria					
Occurrence of malaria					
No malaria history	54.3	1		1	
Had an episode/more	45.7	2044	0.946–4.414	2.205	1.002–4.853
Number of malaria episodes					
1	65	1			
2	12	1.327	0.148–11.888		
3	14	0.724	0.133–3.924		
4	9.0	1	0.104–8.906		
Exposure to Malaria					
No exposure	54.3	1			
Mild	5.0	2.526	0.308–20.709		
Moderate	33.8	2.084	0.882–4.923		
Severe	6.9	1.642	0.346–6.194		
Malaria-related hospitalization					
No	95.8	1			
Yes	4.1	0.325	0.030–3.463		
Malaria during pregnancy					
No	88.6	1			
Yes	11.4	0.731	0.254–2.100		
Malaria attacks during pregnancy					
No	94.1	1			
Yes	5.9	0.4	0.116–1.382		

OR, odds ratio; CI, confidence interval

## Discussion

The findings showed that malaria infection is associated with low cognitive development in children. A survey of 219 children living in a peri-urban area of Manaus showed a high proportion of children with low cognitive development living in poor sanitation conditions, low maternal and family stimulation for learning, with high exposure to malaria. When controlling for potential confounding variables, malaria infections remained as a predictor for low cognitive development in the study population (Table 3).

Studies indicate that malnourished children with high levels of exposure to parasitic diseases [21–23], with low socioeconomic status [24–26], born to young mothers with no schooling [27, 28], are prone to result in poor performance in cognitive tests and language. In a study carried out in Ethiopia, certificated that compared to non-anemic children, anemic children had lower score for the verbal reasoning test [29]. In other study conducted in Peru, found an association between having been infected with ascaris or any STH between one and two years of age and lower cognitive and verbal abilities later in childhood [30].

Favorable environmental conditions, such as adequate stimuli and appropriate family conditions, seem to exert a positive influence on child development [31]. However in this study, no association was found between the cognitive function of the child and the condition of maternal or family stimulation. Possibly, this relationship was not observed due to the low variability of the family stimulus conditions (only 25% are more stimulated) and the mother’s educational level (90% of the mothers lack formal education) in the study population.

The children studied were highly infected with intestinal parasites and were malnourished; 35 (16%) children had anaemia during the period of data collection. The latter condition showed association with low cognitive development (OR 2.245; CI 95% 0.647–7.785). These findings corroborate studies conducted in Indonesia and sub-Saharan African countries that showed the relationship between anaemia (59%), malnutrition (35%) and cognitive development in preschool children [32, 33].

The impact of malaria on childhood cognitive performance has been demonstrated in studies that investigated *P. falciparum* infection [34–36], in which the occurrence of cerebral malaria is more frequent and may result in severe neurological sequelae [37]. Despite the evidence that *P. vivax* malaria can also be associated with neurological impairment [38] and the occurrence of cognitive dysfunction even in uncomplicated malaria [5], little is known about the impact of this infection on cognitive function.

Like other studies that have evaluated cognitive ability in children in Brazil, we found a high proportion (84%) of children with low cognitive development [12, 39]. Vitor-Silva et al., studying children from a rural area of the state of Amazonas where *P. vivax* infection is predominant, showed that the occurrence of at least one episode of malaria throughout the school year was associated with poor school performance (Portuguese or Mathematics subjects) [12].

Brasil et al. found a significant impairment of verbal and full-scale quotients as well as a significant low index of verbal comprehension [40]. The findings corroborate these results, showing that malaria infection is related to the low cognitive development of children in a highly endemic area for *P. vivax* malaria, independent of the socioeconomic status, the family and maternal stimulus, and the child’s health status [39, 41].

Considering that, in the present study, the predominantly affected age group with low cognitive performance was 4–7.7 years old, it is assumed that the impact of malaria on cognitive function is not prolonged. The majority presented with moderate exposure, with episodes up to 2 years or more of age. This result corroborates with that recently described in the literature in which 73% of children in a retrospective cohort study had experienced 1–2 episodes of malaria; 80% of these infections occurred in the previous 2–3 years [35].

Most children in this study only had one documented case of malaria. This is clinically significant in that if only one case of malaria can account for this significant decrease in cognition, it is then extremely important to take the necessary precautionary measures against malarial infection in children. This study argues for further research on the cognitive effects of *P. vivax* malaria infections in children. Further implications for research may lead to applying this same tool to children in other regions of Brazil that are also endemic for *P. vivax* malaria.

## Limitations of the study

The major limitation is that the study is underpowered for multivariate analyses, given the small numbers of children without a history of malaria and the small number of children reportedly without cognitive impairment.

Comparison of this study with others is limited by the fact that this is the only study of its kind to adapt an English cognitive tool for research with Brazilian children and culture. There is no standardized tool which can be utilized globally due to cultural differences [33].

Additionally, children living in *Brasilerinho* generally seem to have low cognitive development as full-scale scores were on average extremely low independent of malaria infection status. This may be due to the fact that many of these children are not in school, as it is believed that it is more important to work, or learn the family trade, than to obtain a formal education.

These findings showed that the occurrence of malaria had a negative effect on cognitive performance in the study population. However, it emphasizes the need for studies with longer follow-up periods. In endemic areas for malaria in Latin America, where *P. viva*x predominates, it is important to include parameters on transmission dynamics of transmission and pathogenesis to predict the real impact of *P. vivax* malaria on cognitive function.

The finding of several conditions that can affect cognitive development reinforces the need to implement practices to assess a child’s cognitive performance within an integrated vision of child health policies. Due to brain plasticity, the child responds better to therapies and stimuli from the environment, especially in early childhood.

## Conclusion

This study has shown that at least one episode of malaria in children results in a decrease in cognition in a *P. vivax*-endemic area in the Brazilian Amazon. In addition to known economic impacts of malaria in the Amazon region, this study highlights the deleterious effects of this endemic on the socio-cultural development of the population.

## Data Availability

The datasets during and/or analyzed during the current study available from the corresponding author on reasonable request.

## Abbreviations

WPPSI-IV: Wechsler Preschool and Primary Scale of Intelligence-IV
qPCR: polymerase chain reaction quantitative
ASD: Autism Spectrum Disorder
ADHD: Attention Deficit Disorder Association
PCR: polymerase chain reaction
DNA: deoxyribonucleic acid
WISC: Wechsler Intelligence Scale for Children
RAF: Resource Inventory Home Environment
BMI: Body Mass Index
STH: soil-transmitted helminthiasis
